# Hypertrichosis and erythrodontia in congenital erythropoietic porphyria

**DOI:** 10.1002/jha2.215

**Published:** 2021-05-26

**Authors:** Leonardo Enciso

**FIGURE 1:**
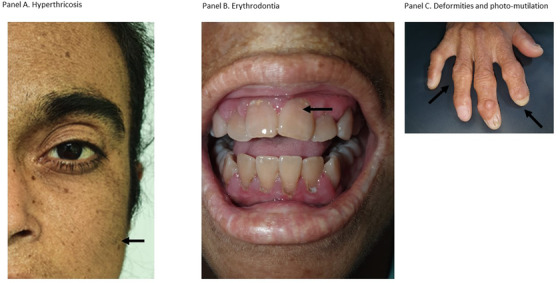
Hyperthricosis, jaundice and Erythrodontia at presentation

A 29‐year‐old female patient was referred to our hematology department for anemia. She was in week 32 of her first pregnancy. Her clinical history was relevant for a diagnosis of porphyria but she does not have medical follow‐up until this pregnancy. The physical examination was relevant for hypertrichosis (Figure 1, panel A), deformities and photo‐mutilation in hands (panel C), splenomegaly, and the presence of erythrodontia (panel B). She has mild hemolytic anemia and reports red urine. All the features were consistent with congenital erythropoietic porphyria (CEP). The pregnancy must end in week 36 because of

premature rupture of membranes. In the follow‐up, sequencing of the UROS gene and a complete biochemical and porphyrins profile were obtained. Total plasma porphyrins level was 1052.3 mcg/L (HPLC normal ranges: 1.0–5.6 mcg/L). The urine porphobilinogen was 1.8 mg/24 h (0–2.5 mg/24 h). The UROS gene sequencing (performed with Illumina TruSight One Expanded kit using NextSeq equipment) found a c.50A>G (p.Asp17Gly) homozygous mutation that confirms the diagnosis of CEP caused by a mutation in the uroporphyrinogen III synthase (MIM#263700). The patient works as a farmer. Avoidance of exposure to visible light with sun‐protective clothing was recommended. Folic acid supplementation was given and continue follow‐up in the ambulatory clinic.

CEP (Gunther disease) is an exceedingly rare disease with less than 300 patients confirmed worldwide. The clinical findings, in this case, are typical of this condition, including hypertrichosis, erythrodontia, hemolytic anemia, and severe skin damage with photo‐mutilation. Usual treatment with sun avoidance measures and skin protection and bone marrow suppression with frequent blood transfusions in cases with severe hemolysis offers partial improvement with hematopoietic stem cell transplantation as the only curative option [1].
